# Cold tolerance response mechanisms revealed through comparative analysis of gene and protein expression in multiple rice genotypes

**DOI:** 10.1371/journal.pone.0218019

**Published:** 2019-06-10

**Authors:** Gabriela Moraes de Freitas, Julie Thomas, Rohana Liyanage, Jackson O. Lay, Supratim Basu, Venkategowda Ramegowda, Marcelo Nogueira do Amaral, Letícia Carvalho Benitez, Eugenia Jacira Bolacel Braga, Andy Pereira

**Affiliations:** 1 Crop, Soil, and Environmental Sciences, University of Arkansas, Fayetteville, Arkansas, United States of America; 2 Department of Botany, Federal University of Pelotas, Pelotas, Brazil; 3 Department of Chemistry and Biochemistry, University of Arkansas, Fayetteville, Arkansas, United States of America; Louisiana State University, UNITED STATES

## Abstract

Due to its tropical origin and adaptation, rice is significantly impacted by cold stress, and consequently sustains large losses in growth and productivity. Currently, rice is the second most consumed cereal in the world and production losses caused by extreme temperature events in the context of "major climatic changes" can have major impacts on the world economy. We report here an analysis of rice genotypes in response to low-temperature stress, studied through physiological gas-exchange parameters, biochemical changes in photosynthetic pigments and antioxidants, and at the level of gene and protein expression, towards an understanding and identification of multiple low-temperature tolerance mechanisms. The first effects of cold stress were observed on photosynthesis among all genotypes. However, the tropical *japonica* genotypes Secano do Brazil and Cypress had a greater reduction in gas exchange parameters like photosynthesis and water use efficiency in comparison to the temperate *japonica* Nipponbare and M202 genotypes. The analysis of biochemical profiles showed that despite the impacts of low temperature on tolerant plants, they quickly adjusted to maintain their cellular homeostasis by an accumulation of antioxidants and osmolytes like phenolic compounds and proline. The cold tolerant and sensitive genotypes showed a clear difference in gene expression at the transcript level for *OsGH3-2*, *OsSRO1a*, *OsZFP245*, and *OsTPP1*, as well as for expression at the protein level for LRR-RLKs, bHLH, GLYI, and LTP1 proteins. This study exemplifies the cold tolerant features of the temperate *japonica* Nipponbare and M202 genotypes, as observed through the analysis of physiological and biochemical responses and the associated changes in gene and protein expression patterns. The genes and proteins showing differential expression response are notable candidates towards understanding the biological pathways affected in rice and for engineering cold tolerance, to generate cultivars capable of maintaining growth, development, and reproduction under cold stress. We also propose that the mechanisms of action of the genes analyzed are associated with the tolerance response.

## Introduction

Climate change can strongly influence agriculture with temperature extremes, cold temperatures being a significant cause of damage in limiting crop yield. Although rice (*Oryza sativa* L.), is one of the world's most important crops consumed as a major part of the diet [1], it is sensitive to cold compared to the temperate crops such as wheat and barley, due to its origin and adaptation for cultivation in tropical and subtropical regions of the world.

The sensitivity and symptoms of plants to cold stress vary with the growth stage. Rice, exposed to cold stress at the vegetative stage, shows symptoms like yellowing of leaves, lower stature, and decreased tillering [2]. Other symptoms include damage to the photosynthetic machinery, more specifically the ultrastructure of chloroplasts, altering the light-harvesting chlorophyll antenna complexes [3] and/or modifying thylakoid structures [4], and an overall reduction in photosynthetic processes by cold temperatures thereby leading to a deficit in plant energy resources.

Under cold stress, reactive oxygen species (ROS) accumulation is induced [5] and can cause severe damage to various cellular components such as altering the membrane lipid composition due to excess accumulation of malondialdehyde (MDA), and an increase in antioxidants that can scavenge ROS and protect rice plants against oxidative damage [6].

In this research report we present results on the physiological responses of a sample of rice *O*. *sativa* sub-species *japonica* genotypes to cold stress, estimated through quantification of photosynthetic parameters, ROS mediated damage, accumulation of antioxidants and osmolytes, that distinguish the sensitive and tolerance rice phenomes. In addition, we present here the analyses of several stress-responsive genes *OsBURB-16*, *OsGH3-2*, *OsSFR6*, *ZFP245*, *OsACA6*, *Ctb1*, *OsSAP1*, *OsTPP1* and *OsSRO1a* (S1 Table) that can potentially contribute towards the observed mechanisms of tolerance.

## Results and discussion

Rice is a major global food crop and a model crop for cereals. To understand the basis of acclimation stability under cold, we used a set of rice genotypes contrasting in their tolerance towards cold and evaluated the photosynthetic, biochemical, gene and protein expression response parameters. The results we describe here suggest the presence of complex mechanisms that involve the interaction of many biochemical and physiological pathways along with hormonal cross-talk contributing to cold tolerance.

The analysis of cold-stress-responsive gene expression at the transcript and protein level, along with the phenotypic response, provides an understanding of cold stress tolerance mechanisms in the multiple plant systems. This information is needed since there is little information available on the signaling pathways responsible for low-temperature acclimation and the differential expression of genes at the transcript and proteins level that provide a crucial role in chilling stress signaling [7,8].

### The response of photosynthetic parameters to cold stress

**Fig 1:**
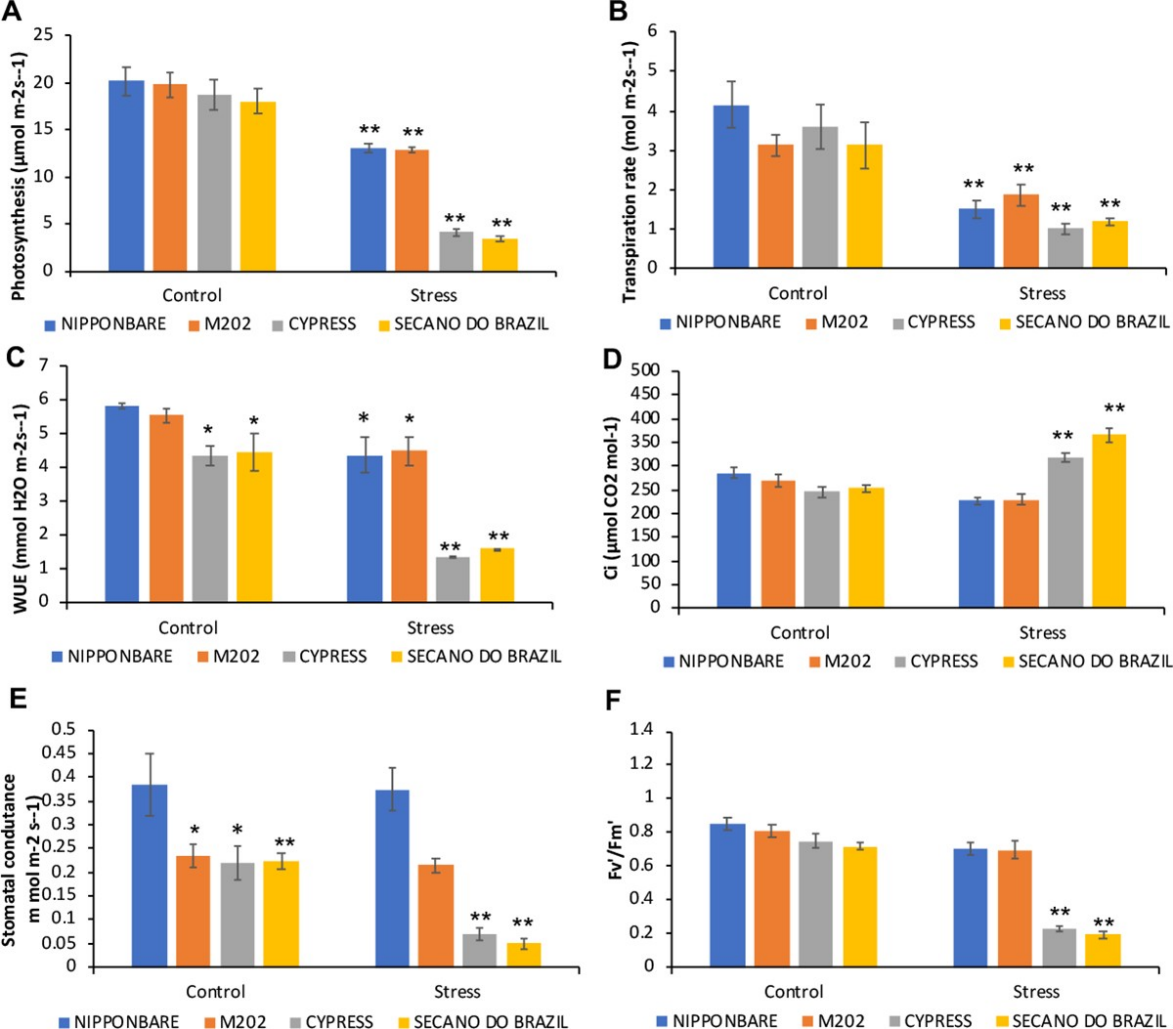
Response in physiological parameters of rice genotypes treated to low temperature stress. Plants under control (28° C) and stress (10° C) conditions show response in: A) Photosynthesis, B) Transpiration Rate, C) Water use efficiency (WUE), D) Intracellular CO2 concentration (Ci), E) Stomatal conductance, F) Fv'/Fm'. Data are expressed as the result of five replications (plants). The asterisks indicate significance at P ≤ 0.01 (analyzed by Student’s t-test) for comparison of stress treatment vs control, and between control plants for difference to Nipponbare, as standard tolerant genotype.

The first effects of cold stress on plants are observed in photosynthesis [9], which was found to be highly affected in the rice genotypes studied (Fig 1A). However, Secano do Brazil and Cypress display > 80% reduction compared to Nipponbare and M202. The reduced air and leaf temperature usually reduce the evaporative demand [10,11], observed as reduced transpiration in all genotypes studied (Fig 1B). However, the tolerant genotypes displayed the highest water use efficiency in comparison to sensitive ones under cold stress (Fig 1C). Under well-watered conditions all the genotypes showed very similar levels but on exposure to stress there was a significant reduction by about 75%.

The chilling stress caused direct effects on stomata, provoking two potential causes of stomatal closure. In Secano do Brazil and Cypress, the direct inhibition of mesophyll photosynthesis (Fig 1A) caused a rise in *c*_*i*_ (Fig 1D), with an associated stomatal closure (Fig 1E) [12,13]. In Nipponbare and M202, the stomata were the primary target of the chilling stress and their closure led to a reduction in *c*_*i*_, prompting a decline in photosynthesis [10,14]. Photo-inhibition can be one of the primary causes of reduction in photosynthesis after cooling [15–16] and is characterized by a reduction in Quantum Efficiency of PSII [17]. A significant reduction was seen in Secano do Brazil and Cypress under stress, but for the same conditions, the tolerant genotypes did not suffer photoinhibition (Fig 1F). The fluorescence parameter Fv’/Fm’ is regarded as a suitable assay for plant tolerance and sensitivity to cold [18], due to inherent tolerance or physiological acclimation.

### Biochemical parameters affected by cold-stress

**Fig 2:**
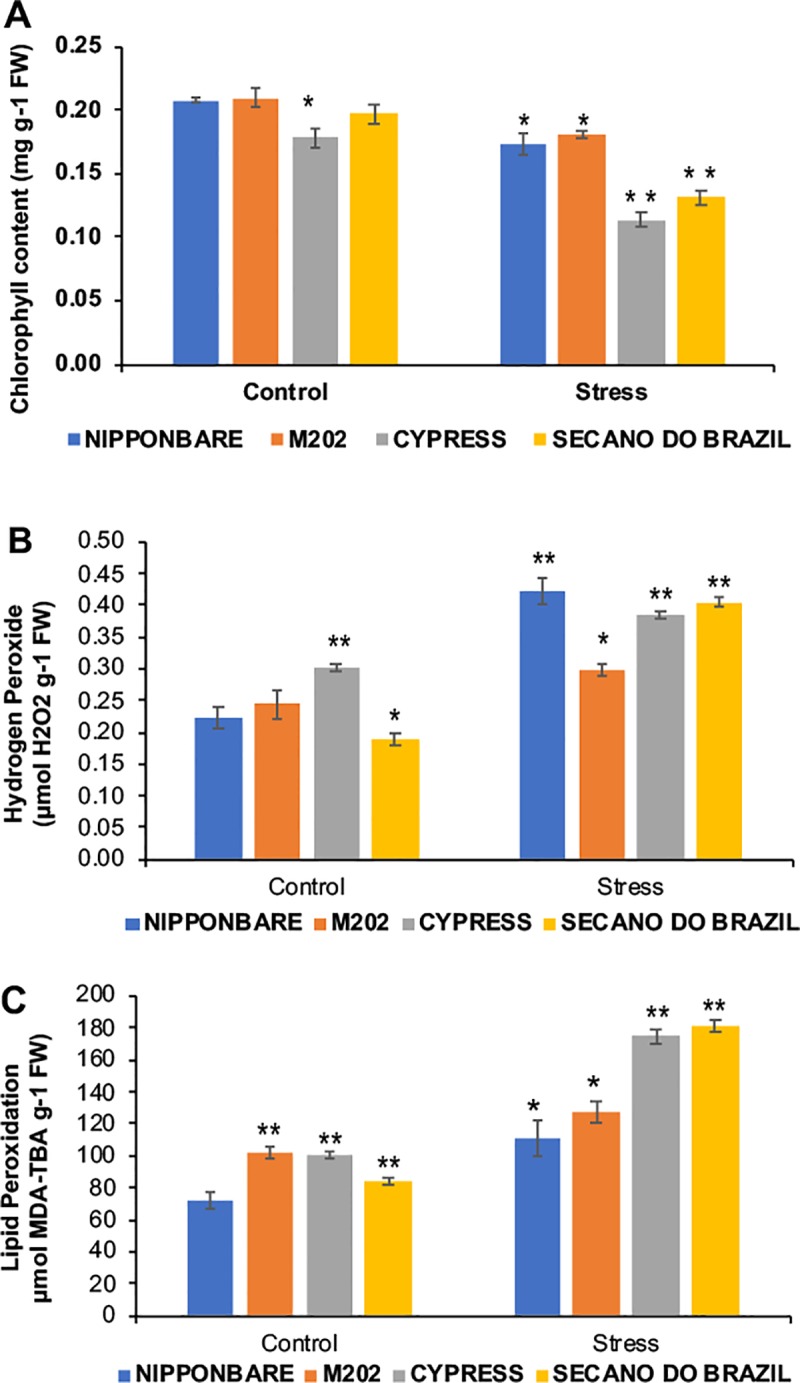
ROS mediated damage evaluated in different rice genotypes treated to low temperatures. Plants under control (28° C) and stress (10° C) conditions showed response in: A) Chlorophyll content, B) Hydrogen peroxide H_2_O_2_ and C) Lipid peroxidation MDA. Data was expressed as the mean of five replications. The asterisks indicate significance at P ≤ 0.01 (analyzed by Student’s t-test) for comparison of stress treatment vs control, and between control plants for difference to Nipponbare, taken as standard tolerant genotype.

When plants are exposed to low-temperature stress, Chlorophyll biosynthesis is affected (Fig 2A), and the results of the experiment show that Nipponbare and M202 do not sense the stress like the sensitive genotypes Secano do Brazil and Cypress, which show a significant impact on the biosynthesis of Chlorophyll (Fig 2A). According to [17], this is because the impact on Chlorophyll biosynthesis is due to down-regulation of gene expression and protein abundance of several enzymes involved in tetrapyrrole metabolisms described in other studies [19,20,21].

**Fig 3:**
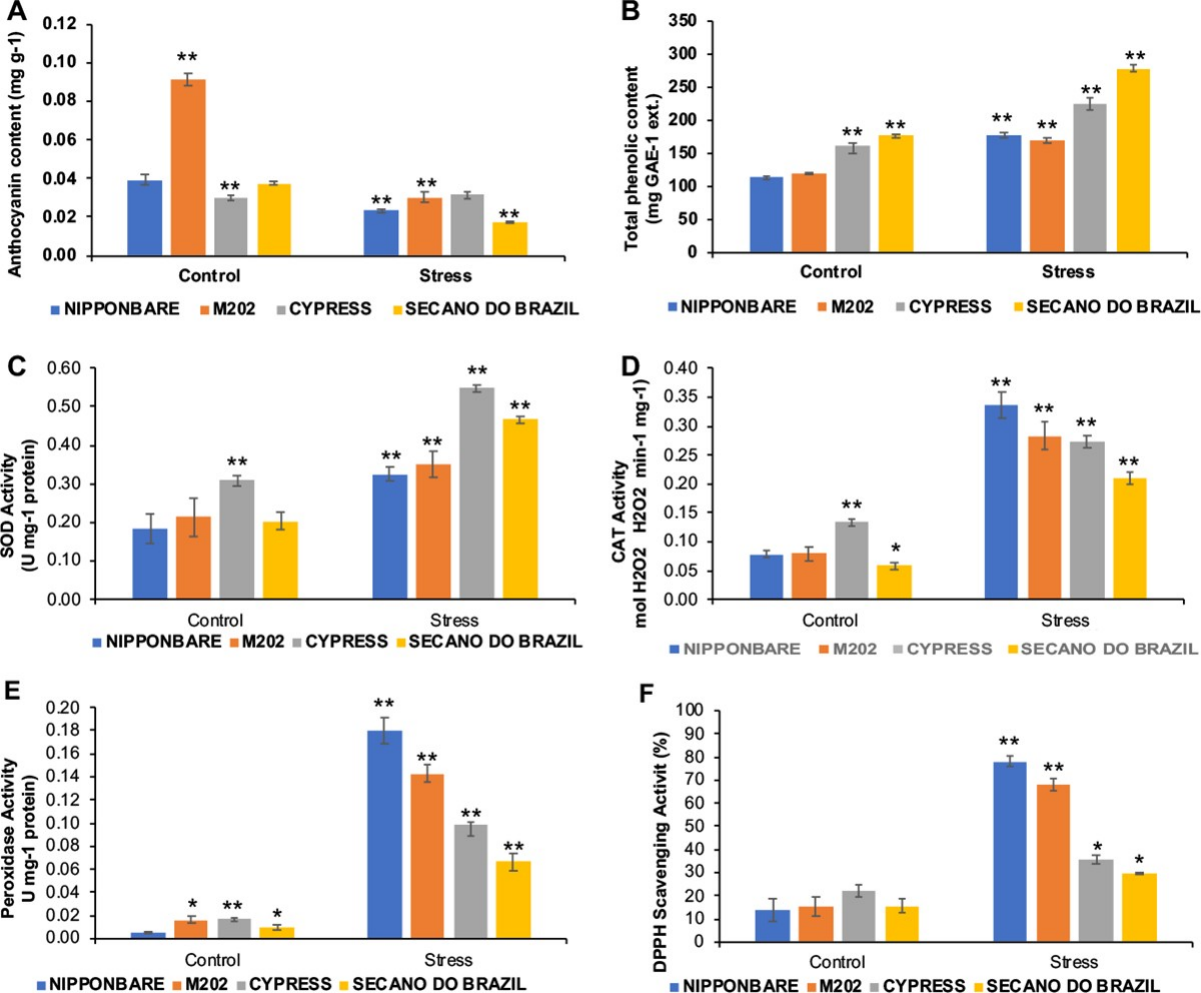
Non enzymatic antioxidants and enzymatic antioxidants were evaluated in different rice genotypes treated to low temperatures. Plants under control (28° C) and stress (10° C) conditions showed response in: A) Anthocyanin content, B) total phenolic content, C) superoxide dismutase (SOD) activity, D) catalase CAT activity, E) Peroxidase activity, and F) 2,2-diphenyl-1-picrylhydrazyl-DPPH activity. Data are expressed as the result of five replications. The asterisks indicate significance at P ≤ 0.01 (analyzed by Student’s t-test) for comparison of stress treatment vs control, and between control plants for difference to Nipponbare, taken as standard tolerant genotype.

The anthocyanins, which are induced by environmental stresses [22], have a role in modifying the quantity and quality of captured light [23], by protecting from the effects of UV‐B [24], and scavenging of reactive oxygen intermediates under stress. However, our data showed a significant reduction in M202 by about 50% and by about 75% in Secano do Brazil, while Cypress showed no difference between the control and stressed plants (Fig 3A). These results do not support the hypothesis that anthocyanins are needed to serve an auxiliary photo-protective role in leaves, because this variation is independent of the concentrations of chlorophyll (Figs 2A and 3A). However, the anthocyanins are found predominantly associated with leaf mesophyll [25], a location that is unsuitable for screening out UV-B but ideal for the scavenging of oxygen radicals produced by chloroplasts. The phenolic content showed an increase in all genotypes, with the highest content in the sensitive genotypes Secano do Brazil and Cypress (Fig 3B). This increase may help in restricting the penetration of UV-B into the inner tissues of the plant [26], and contribute to their antioxidant ability, which inhibits lipid peroxidation by trapping the lipid alkoxyl radicals [27,28].

**Fig 4:**
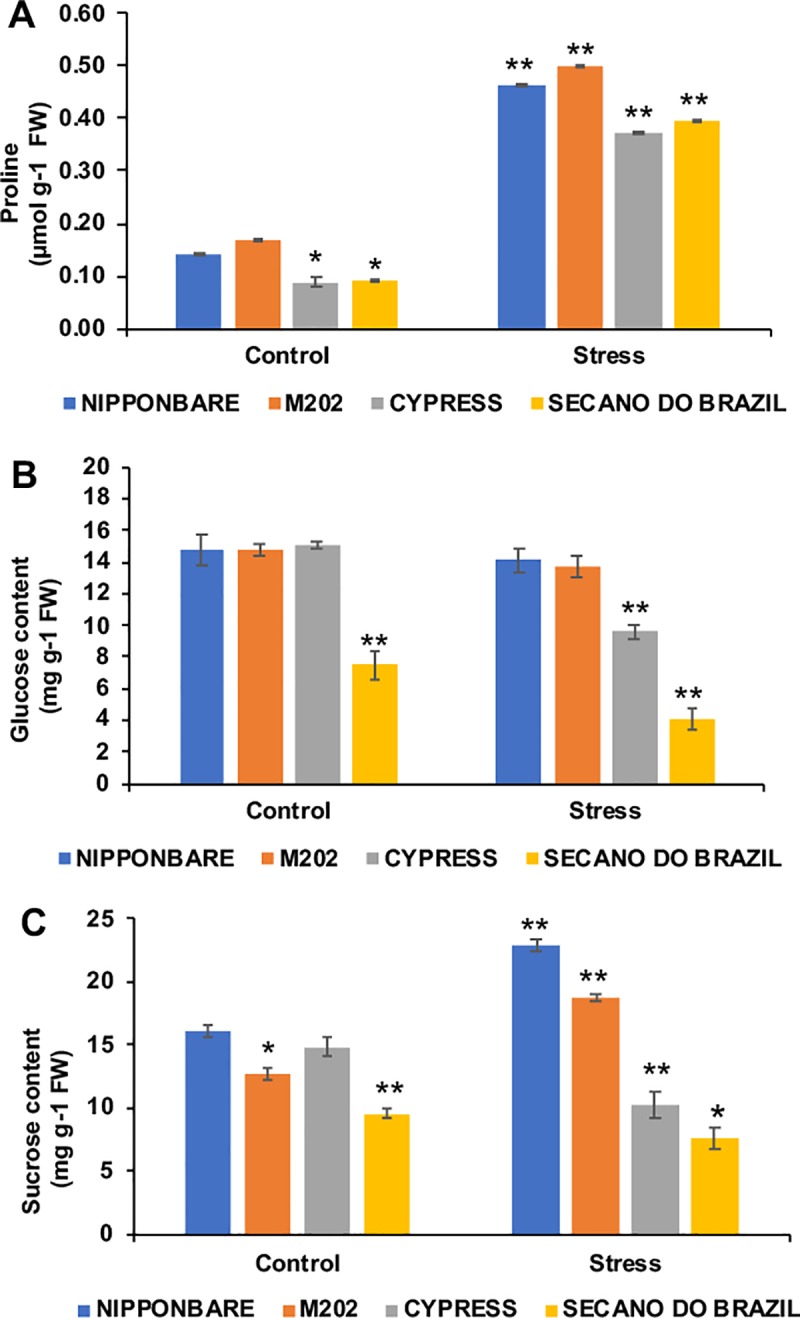
Response of rice plants to temperature treatments, with control (28° C) and stress (10° C) conditions showing differential response in osmolyte content. A) Proline, B) Glucose content and C) Sucrose content. Data are expressed as the mean of five replications. The asterisks indicate significance at P ≤ 0.01 (analyzed by Student’s t-test) for comparison of stress treatment vs control, and between control plants for difference to Nipponbare, taken as standard tolerant genotype.

Proline and soluble sugars are also known to protect rice from damage due to cold stress [29]. Proline content was found to be enhanced by cold stress in all genotypes (Fig 4A), with the tolerant lines showing a higher synthesis in control and stress conditions, with results similar to that obtained earlier [30]. Proline is involved in the removal of stress-related excess H^+^, maintains oxidative respiration at optimal cytosolic pH [31], acts as a reservoir of carbon and nitrogen, and increases protein water-binding ability through its hydrophobic interactions with the surface residues of proteins [32].

Soluble sugars like glucose and sucrose can accumulate in plants under stress, and act as osmoprotectants against freezing/dehydration damage as described previously [33]. The tolerant genotypes (Nipponbare and M202) showed no significant difference in glucose content, although the sensitive genotypes showed a decrease (Fig 4B). In contrast, the sucrose content showed a high increase in the tolerant genotypes compared to the control, while for the sensitive genotypes a significant drop was observed (Fig 4C). Such an increase in sucrose has been documented previously [34], suggesting that an increase under low temperature can be a useful marker for cold tolerance in rice.

Many plant subcellular locations such as the cell wall, plasma membrane, mitochondria, and chloroplast, at the site of thylakoid electron transport; and the nucleus are intrinsically responsible for producing ROS in response to stress [35,36,37]. To avoid disastrous damage to protein and lipid components, plants have numerous antioxidant systems. Among these, ROS (e.g. H_2_O_2_) can cause cellular and tissue damage by degradation of polyunsaturated lipids to form MDA, which is a reactive aldehyde causing toxic cellular stress [38]. Nipponbare, despite showing the highest H_2_O_2_ production under stress (Fig 2B), showed the lowest damage on the plasma membrane (Fig 2C); whereas the tolerant M202 under stress, with no significant H_2_O_2_ induction, showed damage with MDA induction. In contrast, the sensitive genotypes had high production of H_2_O_2_ and MDA, causing a great impact on the cell membranes, as shown previously [39].

The antioxidant enzymes SOD, POD and CAT, present in tolerant genotypes can compete against ROS formation [40], providing antioxidant activity to offer protection from oxidative stress damage [41].These enzymatic mechanisms, found among plants challenged to cold and other abiotic stresses, function in ROS scavenging by the reduction of superoxide radicals into H_2_O_2_ as analyzed here for the SOD (Fig 3C), CAT (Fig 3D) and POD (Fig 3E) expression activities that catalyze H_2_O_2_ into H_2_O and protect the plant cells from H_2_O_2_ accumulation [42]. The bulk of H_2_O_2_ from SOD catalysis remains biologically toxic. SOD activity observed under stress (Fig 3C) in cold tolerant genotypes, can limit plant damage from ROS, and is lower in cold-sensitive genotypes. H_2_O_2_ production by SOD enzymes can also function in oxidative stress signaling, to play the role of a secondary messenger and protect reactions leading to induced CAT and POD activity in plants [43]. Biochemical analysis of cold-sensitive genotypes revealed that the lower increase of CAT and POD activity could reduce the efficiency of the plant cells to scavenge damaging free radicals. The analyses suggest that most of the plant’s response in increasing antioxidant activity has an important role towards cold stress tolerance. The high stability and increased rate of CAT and POD activity are known to confer cold-induced oxidative stress tolerance [44,45].

DPPH application was used to measure free radicals as a measure of stress tolerance [46]. The increase in DPPH radical scavenging (Fig 3F), observed in Nipponbare and M202, also appears to be correlated with the degree of plant stress tolerance [47,48]. Cold tolerance has been quantified by measuring the reduction in growth rate and cell membrane stability [49]. The DPPH assay reveals a higher antioxidant capacity in the tolerant genotypes (Fig 3F), supporting the relationship between antioxidant capacity and cold tolerance. The observed relationship between CAT and POD activity, and cell membrane stability, supports the importance of sustaining an optimum antioxidant content under stress for the expression of cold tolerance. Cold-tolerance in plants is generally associated with a higher antioxidant capacity that is induced under stress, compared to the response of cold-sensitive plants [50].

### Gene expression responses to cold-stress treatment

**Fig 5:**
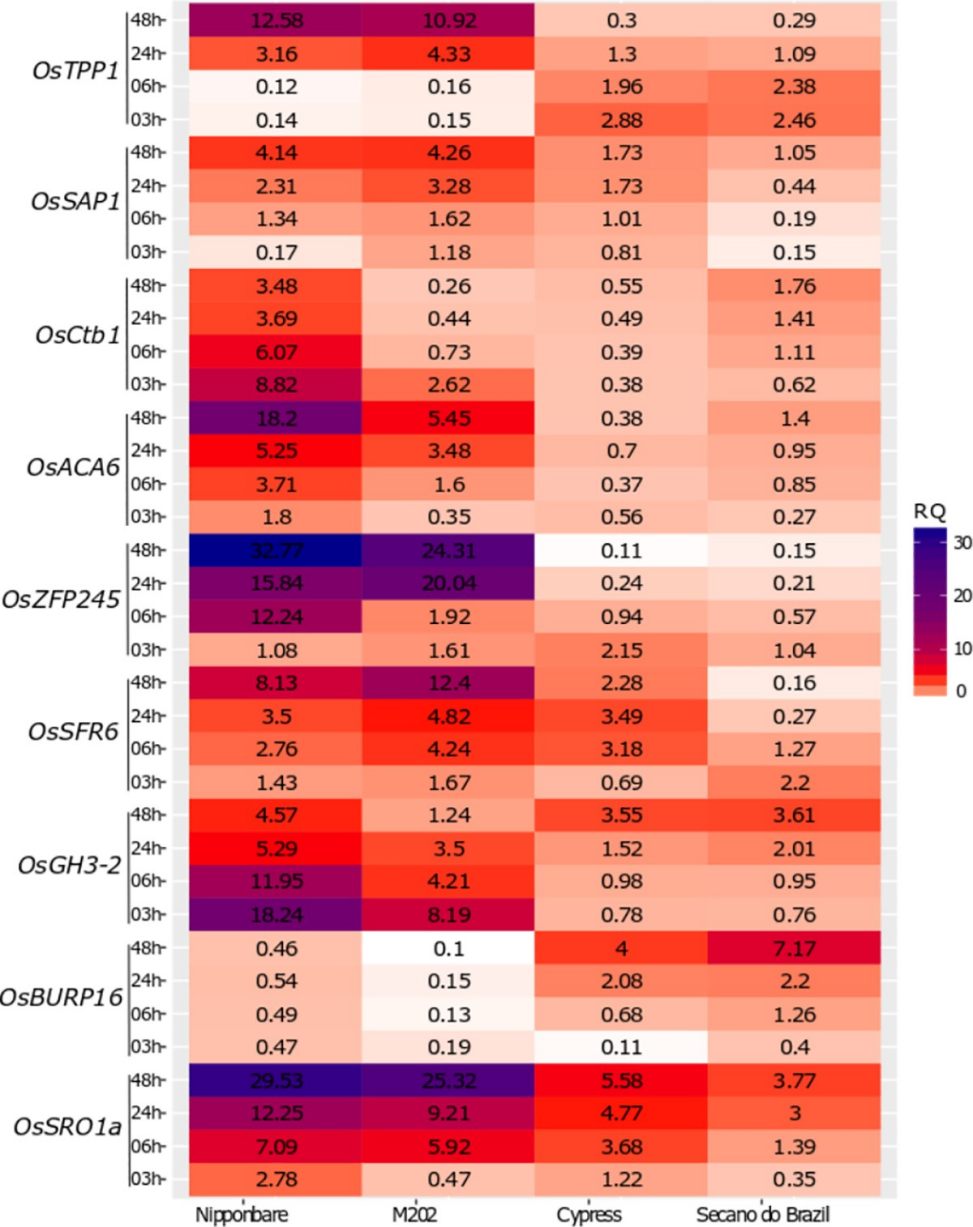
Relative expression of genes (S1 Table) conferring stress tolerance to low temperatures in rice. Stress tolerance related genes are shown in a time-course of 03–48 h after stress initiation. Data are results from three biological replicates and are expressed as the relative quantification (RQ) ratio of fold change of stress treatment to control.

To support our studies on phenotypic and biochemical responses to cold stress of tolerant and sensitive rice genotypes, a bibliographic review was conducted and identified several stress-responsive genes under low-temperature conditions. From this literature, we selected nine cold-regulated genes, and their expression behavior was characterized in the four different genotypes at different time points after stress (3h, 6h, 24h, and 48h) in the vegetative plant growth stages (Fig 5). Cold acclimation can involve alterations in gene expression and changes in the levels of particular proteins following cold treatment [51].

The plant cell wall, which can be considered a layer to safeguard against abiotic stress such as cold [36], is a complex structure inserted in a physiologically active pectin matrix, cross-linked with structural proteins and, depending on the tissue/organ, with lignin [52,53]. The *OsBURP16* gene, encodes a putative precursor of PG1β, a subunit that regulates the activity of polygalacturonase (PG), an enzyme which hydrolyzes pectin, and changes the composition of the plant cell wall [54]. Our data showed that the expression of *OsBURP16* was induced maximally in sensitive genotypes (Secano do Brazil and Cypress) and had an increased transcript level with time, while Nipponbare and M202 had little variability in the same period (Fig 5). Therefore, the *OsBURP16* gene can be considered as an indicator of cold stress sensitivity [54]. Unlike *OsBURP16*, *OsACA6* exhibited an increased level of expression in tolerant genotypes and Secano do Brazil (sensitive), however, Nipponbare had a higher expression. Located in the plasma membrane are pumps or antiporters, which act on the cold response pathway involving the influx of Ca^2+^ from the apoplast to cytosol [55].

Under cold stress, plants exhibit a decrease in H_2_O_2_ and MDA content, with an increase in membrane stability, CAT, SOD and APX expression [35], and proline content [29]. These changes have been shown to indicate a positive response of cold tolerance in transgenic tobacco [56].

The *OsGH3-2* and *Ctb1* genes show early and maximal induction after 3 h of stress and decrease with time in the tolerant Nipponbare and M202, while in Cypress and Secano do Brazil there is an increase in expression with time (Fig 5). The overexpression of *OsGH3-2* [57], has been shown to decrease drought resistance and stomatal closure, as well as increase water loss and improvement of cold and oxidative stress tolerance in rice at the vegetative stage. On the other hand, [58] report that in association with CAT, *Ctb1* participates directly in the regulatory pathway of small-RNAs and promotes cold-tolerance at the reproductive stage.

*OsSRO1a* and *OsSAP1* were quickly induced after 3 h, reaching a maximum at 48 h in Nipponbare and M202 (Fig 5). For the sensitive genotypes, we found the same pattern, although the expression was lower. SRO are proteins involved in ADP-ribose conjugation, DNA repair, apoptosis, transcription, and chromatin remodeling [59]. They possess a C-terminal RCD1-SROTAF4 domain and interact with *AP2/EREBP* and transcription factors *OsDREB2A* [60]. In rice, *OsSRO1* has a role in drought and oxidative stress tolerance, stomatal closure and H_2_O_2_ accumulation [61]. *SAPs* can interact with proteins via their zinc-finger domains [62], such as *OsSAP1* with cytoplasmic kinase *OsRLCK253* [63]. *OsSAP1* can regulate the stress responses by either modulating the expression of genes or by interaction with other proteins [64]. Altogether, our results suggest that this interaction may have relevance in stress physiology and cold-acclimation.

Trehalose-6-phosphate phosphatase (TPP) is a sugar storage metabolic regulator and acts in protection against abiotic stress [65,66]. In rice, overexpression has shown increased tolerance to abiotic stresses [67,68]. Our data showed that the *OsTPP1*gene is induced after 24 h and increases more at 48 h in the tolerant genotypes, while Cypress and Secano do Brazil show a slight induction at 3 h that goes down completely after 48h (Fig 5). These results agree with other studies [69], that show overexpression of *OsTPP1*is enhanced in salt and cold tolerance of rice. Moreover, they also showed that in plants with high expression of *OsBURP16* there is a decrease in trehalose content.

Among the genes studied for expression under cold stress, *OsZFP245* and *OsSFR6* showed the highest induction in the tolerant genotypes, with the peak induction at 48 h. In Cypress, *OsSFR6* showed increased expression unto 24 h. However, in the sensitive Secano do Brazil both genes (*OsZFP245* and *OsSFR6*) were induced early with maximal expression at 3 h, and sensitive Cypress induced *OsSFR6* early with the highest expression at 24h (Fig 5). Located in the nucleus, *OsSFR6* acts to induce Cold-On Regulated (COR) genes via transcription factors CRT⁄DREs 2A and CBFs 1–3 [70], and *OsZFP245*, a zinc finger protein gene with the role of increasing proline content and antioxidant enzymes [71,72].

**Fig 6:**
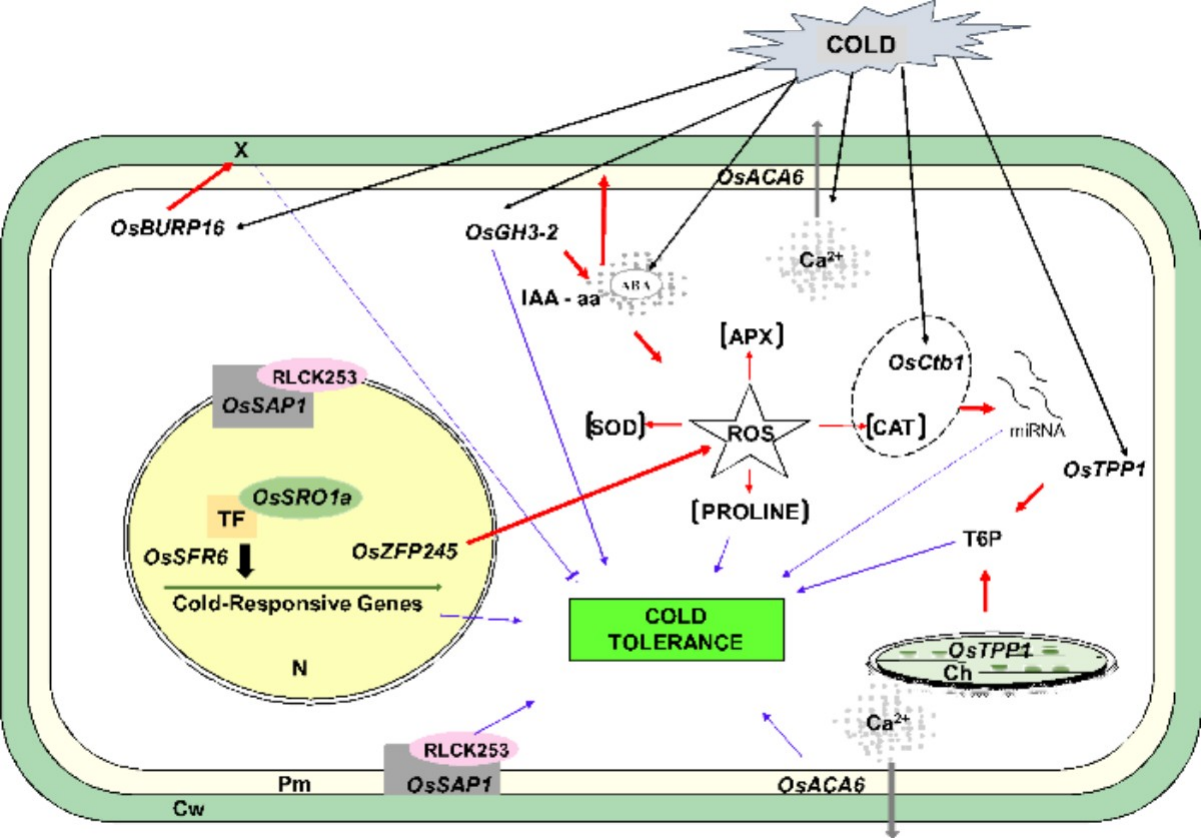
Model displaying mechanisms of tolerance to low temperatures in rice. The regulatory cascade indicates the perception and induction of damage in response to low temperatures, the response in gene expression changes to the stress treatment, as well as the induction of biochemical responses (S3 Table) leading to low temperature tolerance, with an increase in concentration due to the presence of ROS. Abbreviations shown indicate the changes in components affected. **Pm**: Plasma membrane; **Cw**: Cell wall; **Ch**: Chloroplast; **N**: Nucleus; **Grey arrow**: Calcium efflux **Black arrows**: Cold perception; **X**: Degradation of pectin caused by increased polygalacturonase induced by increased expression of *OsBURP16*; **Red arrow**: Induction; **Blue arrow**: Induction of cold tolerance; **Between brackets**: Increase in concentration due to ROS. **Dotted**: Association of *Ctb1* and CAT for miRNA induction. **Upward arrows**: Increased concentration due to induction of ROS; **X**: Association of *Ctb1* and CAT for miRNA induction. The regulatory cascade of perception and induction of damage in response to low temperatures, and response of genes to the stress treatment, as well as the induction of biochemical responses leading to tolerance to low temperatures, leading from an increased concentration of ROS.

A model for low-temperature tolerance mechanisms (Fig 6) was developed by integrating information on gene expression response to cold in the genotypes, to improve our understanding of how stress is perceived by cells and how the regulatory cascade of signals act to promote tolerance to suboptimal temperatures.

Stress is perceived by cells through molecular signals, which in turn, induce a concomitant expression of multiple genes (cited in the text). They act in the perception and induction of damage caused by suboptimal temperatures generating responses that may, for example, increase the activity of antioxidant and osmoprotectant enzymes, and result in greater tolerance to stress.

### Differential protein expression under cold-stress

To improve our understanding on the response of rice plants to cold stress, we used a comparative proteomics approach to study the effect of cold stress on rice genotypes differing in tolerance and observed differential protein expression between tolerant and sensitive genotypes (S1 Table).

The leucine-rich repeats protein kinases (LRR-RLKs) play an important role in regulating plant responses to abiotic stress [73], supported by our data that shows tolerant genotypes have higher expression compared to the sensitive. These results were also observed earlier [74], which supports that *GsLRPK* increases kinase activity in the presence of cold stress and increases expression of low-temperature- responsive genes, resulting in an enhancement in the tolerance to cold stress. Other kinases like the Fructokinase-2, Phosphoribulokinase, and Nucleoside diphosphate kinase 1 genes are also regulated by environmental stresses and show the same expression behavior in tolerant genotypes.

GF14 shows significant homology with protein kinase-dependent regulatory proteins [75]. In rice, the 14-3-3 proteins GF14b, GF14c, GF14e and GF14f, interact with target proteins that are involved in stress response [76]. GF14-a showed low expression in sensitive genotypes and high expression for GF14-f, suggesting that they may be involved in mechanisms of tolerance and/or acclimatization to adverse environmental conditions.

The bHLH proteins are a group of transcription factors that carry out key roles in phytochrome signal transduction, cell fate determination and stomatal differentiation [77]. These transcription factors are stress-inducible under drought, cold or high-salinity and are brassinosteroid-responsive[77,78], and this inductive behavior was also observed in the cold tolerant M202 and Nipponbare genotypes.

In plants, methylglyoxal a by-product of glycolysis, is toxic and causes damage to cells, and high cellular concentrations are generated from unfavorable environmental conditions [79,80]. Glyoxalase proteins are very important for limiting methylglyoxal levels, and for this, the plants have a glyoxalase system with multiple isoforms of both GLYI and GLYII proteins[81,82,83]. Due to its role in methylglyoxal detoxification, over-expression of glyoxalase system in plants confers significant tolerance against adverse environmental conditions [79,84,85,86,87,88]. Evidence of increased tolerance was demonstrated by elevated protein expression (Putative glyoxalase I) in the tolerant genotypes studied.

Lipid transfer proteins (LTPs) play an important role in abiotic stress tolerance [89], and can facilitate the inter-membrane exchange and transfer of various amphiphilic molecules including phospholipids, glycolipids, steroids, acyl-CoAs, and fatty acids [90]. Transcript levels of LTPs increased in response to drought [91], salt [92] and cold [93] and the increase of non-specific lipid-transfer protein 1 expression in tolerant genotypes give more support to its role of increasing tolerance to abiotic stress.

## Conclusions

This study supports that the genotypes Nipponbare and M202 have tolerance to low temperatures with the evidence of physiological responses, such as photosynthesis showing lower reduction, better efficient use of water without suffering photoinhibition, or reduction in the Quantum Efficiency of PSII. The biochemical profile showed that for the same genotypes, chlorophyll biosynthesis was not affected. Among the anthocyanins, a significant decrease in their content was observed, which identified pigments associated with the leaf mesophyll that act directly in the elimination of oxygen radicals produced by the chloroplasts. Accumulation of proline, glucose, and sucrose was also observed, these being osmoprotectants against freezing and dehydration damage. Antioxidants in the same tolerant genotypes, despite showing high production of H_2_O_2_ under stress, did not cause a high impact on the plasma membrane or the high activity of the antioxidant enzymes. SOD, CAT, POD and DPPH enzymes play an important role in stress tolerance. Differential expression of genes and proteins: the genes *OsGH3-2*, *OsSRO1a*, *OsZFP245* and *OsTPP1*; and the LRR-RLKs, BHLH, GLYI, and LTP1 proteins, showed a clear difference in expression between tolerant and sensitive, thus suggesting that these genes are good candidates for identification of low-temperature tolerant genotypes in rice that are capable of maintaining growth, development, and production at the desired agronomic levels. Finally, based on our studies, a schematic representative model of cold tolerance in rice (Fig 6) is proposed outlining mechanisms of action of the genes analyzed with differential responses in resistant genotypes, with the objective of improving our understanding of the operation of tolerance to low temperatures. To summarize the results, our analysis shows for the first time the role of different antioxidants and osmolytes in modulating the physiological responses contributing to tolerance. In addition, this report also identifies markers for screening of cold tolerance in multiple rice genotypes, along with few putative protein markers identified from LCMS/MS analysis.

## Materials and methods

### Plant growth conditions and cold stress treatment

Seeds of the genotypes temperate *japonica* Nipponbare and M202 (tolerant), and the tropical *japonica* Cypress and Secano do Brazil (sensitive) from the USDA mini-core collection [94] were sterilized, immersed in deionized water and germinated in the dark for five days. After germination, seedlings of the same size were transplanted into 500 ml capacity plastic pots filled with commercial substrate (Redi-earth) with known water retention capacity and weight. All the individual genotypes in the pots were grown in a Conviron growth chamber set at26/22° C ±1°C day/night temperature and light intensity of 600 μmolm^-2^s^-1^, with day/night cycle of 14/10h, and kept in trays filled with water simulating flooded conditions with periodic commercial fertilization using Miracle-Gro (Scotts Miracle-Gro Products) for the entire period of the experiment.

Fifteen days after transplanting, the uniformly growing plants were divided into two groups with 20 pots each, five pots per genotype, each containing one plant. One group was kept under ideal conditions (28° C) and served as control, while another set of trays was used for low treatment temperature (10° C) as described [95] with adaptations. For both treatments, the plants were kept under the conditions described for a period of 72h. For this experiment, the experimental design was a complete randomized design and the data were submitted to analysis of variance (ANOVA) and Student's t-test (1%), using the SAS 9.3 statistical program (SAS Institute, Cary, NC).

For the analysis of gene expression, 2 cm leaf tissue fragments were collected at four times 3h, 6h, 24h, and 48h, after low-temperature initiation, while the samples for the biochemical and proteomic analyses were collected only at 72 hours. The photosynthesis and photochemical efficiency of PSII were evaluated using the second fully expanded leaf at 72h using a portable photosynthesis meter (LI-6400XT; LI-COR) at a CO_2_ concentration of 370 μmol mol^-1^light intensity of 1,000 μmol m^-2^ s^-1^ and 55% to 60% relative humidity.

### Biochemical analysis

Chlorophyll content was estimated following the method of [96], using absorbance for chlorophyll a at 663 nm and chlorophyll b at 645 nm, that was measured with a UV-Vis spectrophotometer. The hydrogen peroxide content was determined as described [97], with absorbance at 390 nm. Malondialdehyde (MDA) Buffer solution was made with 0.07% NaH_2_PO_42_H_2_O and 1.6% Na_2_HPO_4_, 1% H_2_O_2_, and 20% trichloroacetic acid containing 0.5% thiobarbituric acid. The absorbance of the supernatant was read at 532nm and MDA concentration was calculated using the MDA extinction coefficient of 155 mM^-1^cm^-1^ [98]. Anthocyanin was quantified as described [99], the absorbance measured at 525 nm and the anthocyanin concentration calculated in mg/gm of fresh weight using the millimolar extinction coefficient of 31.6. The content of phenolics in the extract was determined according to [100] with some modifications, with absorbance measured at 760 nm.

For the determination of Superoxide dismutase (SOD), Catalase (CAT) and total Peroxidases (POD), the total proteins were extracted as described [101]. Total Superoxide dismutase activity, the basis of its ability to inhibit the photochemical reduction of nitroblue tetrazolium (NBT) [102], was assayed as described [103] with some modifications, the absorbance of the reaction mixture measured at 560 nm. Catalase enzyme activity was assayed as described [104], with the decrease in H_2_O_2_ assayed by a decrease in optical density at 240 nm, and the activity calculated using the extinction coefficient of 40 mm^-1^ for H_2_O_2_. The peroxidase (POD) activity was determined using the method of [105].

The antioxidant activity of the extracts, based on the scavenging activity of the stable 1,1-diphenyl-2-picrylhydrazyl (DPPH) free radical, was determined following [106]. The absorbance at 517 nm was used to calculate [(A_0_-A_1_)/A_0_] x 100 where A_0_ is the absorbance of the control, and A_1_ is the extract. Glucose was estimated using 3,5-dinitrosalicylic acid (DNS) according to the method described [107]. The absorbance was recorded at 570 nm where glucose served as the standard. The sucrose was estimated using the method described by [108], and the absorbance was recorded at 620 nm. Free proline content was determined according to the procedure of [109], and the red color intensity was measured at 520 nm.

### Analysis of Gene expression

Total RNA was extracted, using Trizol reagent (Invitrogen), from rice genotypes and cDNA was synthesized using 2 μg total RNA treated with DNAse using GoScript reverse transcription system (Promega). RT-qPCR reactions were performed using GoTaq qPCR Master Mix (Promega) with Ubiquitin as an internal reference gene [110]in a 96-CFX thermocycler(Bio-Rad).The temperature increase (0.5° C 10 s^-1^) from 55°C to 95°C was used for the analysis of the melting curve. Non-transcribed RNA was also run as a negative control. For qPCR analysis the primers were selected based on literature searches on their role in cold tolerance and sequences derived from accession numbers obtained from rice genome databases were used for primer designing using IDT primer designing tool. The primers used are listed in S2 Table. The relative expression differences for each of the samples in individual experiments was determined by normalizing the Ct value for each gene in relation to Ubiquitin Ct value and the relative fold change was calculated using the equation 2^-ΔΔCt^ [111]. The expression analysis was performed with three biological replicates and two technical replicates. Total RNA isolated from three different leaves collected from three different plants under control and treatment are treated as biological replicates while an aliquot of same sample for each sample was duplicated serving as technical replicates. Data were used in the analysis of variance (ANOVA) and Student's t-test (1%), using the statistical program SAS 9.3 (SAS Institute, Cary, NC).

### Analysis of protein expression

Total protein of four rice genotypes used in this work was extracted using the Protein Isolation Buffer and methodology as described by [95]. The protein concentration in each sample was determined by the Bradford assay [112] using bovine albumin as the standard (Fraction V, Sigma). Total protein samples were loaded onto SDS-PAGE-Gel, with samples of 90 μg of protein. Spots of interest, showing differences were excised from the gel and digested using the protocol described by [113]. All MALDI-MS and MS/MS analyses were performed using Ultraflex II MALDI-TOF/TOF mass spectrometer (Bruker Daltonik, Bremen, Germany). All LC-MS/MS was performed using Bruker Amazon-SL quadrupole ion trap mass spectrometer with a captive spray ionization source. The resulting LC-MS/MS spectra were analyzed by Skyline-daily 3.6.9 software and shown in S1 Table [114].

## Supporting information

S1 Table Genes with accession numbers, forward and reverse primer sequences, and efficiency. (PDF)

S2 Table Summary of proteins identified by MS/MS (MALDI TOF/TOF) differentially expressed between the tolerant (Nipponbare and M202) and sensitive (Secano do Brazil and Cypress) genotypes in response to cold stress. (PDF)

S3 Table Quantification of biochemical changes under cold stress. (XLSX)

S4 Table Quantification of whole plant gas-exchange physiological changes under cold stress. (XLSX)

S5 Table Quantitative PCR results of stress responsive differentially expressed genes. (XLSX)
