# Supplementary material for: Cold tolerance response mechanisms revealed through comparative analysis of gene and protein expression in multiple rice genotypes
Source: PLoS One. 2019 Jun 10;14(6):e0218019. doi: 10.1371/journal.pone.0218019 (PMC6557504; doi:10.1371/journal.pone.0218019)
Supplement: S1 Table — (PDF) [file pone.0218019.s001.pdf]

Supplementary Table 1. Genes with Accession Numbers, Forward and Reverse Primer Sequences, and Efficiency

**Gene**	**Locus**	**F: Primer (5'-3')**	**R: Primer (5'-3')**	**Efficiency**
OsBURP16	LOC_Os10g26940	CCTCTGCCCTAACTCTACCTAT	TCGTATGTGAAGGTGCATGAG	1,99
OsGH3-2	LOC_Os01g55940	TGACACTGACACCGACTG	ATGCTTCACCACATCATT	2,10
OsSFR6	LOC_Os10g35560	CGGTGGTGACTAAGTGGTTGTC	GTACTAGAGTTTGCAGGAAGCCAT	2,00
OsZFP245	Os07g0588700	GGAGGCTCTGTCAAGGAGAA	GTGAGCTCTCAGCCTCGTCT	1,85
OsACA6	LOC_Os01g71240	GAAGGTGTCCATCTACGACATC	CGACGAACGAGTATCCATCAA	2,08
OsCTb1	LOC_Os03g21060	GGCTCTCATCTGGTTTCTTCATGG	ACGAAAAGGGGGGTGTCATTTTTC	2,02
OsSAP1	LOC_Os06g41010	TTTTAATTGCAAACGGGAGGATA	TCGATTCTTTTTCCCTCAACCA	1,97
OsTPP1	LOC_Os02g44230	TGCCTCCACACCTGCAGTGACAAG	GCAAGAGACCGGATCCACCATCACTG	1,99
OsSRO1a	LOC_Os01g42860	TCCAATTCCCTGTGCTTGAAG	CTTGCCAGTGTCATCAAACCA	2,05
