# Supplementary material for: Cold tolerance response mechanisms revealed through comparative analysis of gene and protein expression in multiple rice genotypes
Source: PLoS One. 2019 Jun 10;14(6):e0218019. doi: 10.1371/journal.pone.0218019 (PMC6557504; doi:10.1371/journal.pone.0218019)
Supplement: S2 Table — (PDF) [file pone.0218019.s002.pdf]

**Supplementary Table 2.** Summary of proteins identified by MS/MS (MALDI TOF/TOF) differentially expressed between the tolerant (Nipponbare and M202) and sensitive (Secano do Brazil and Cypress) genotypes in response to cold stress.

| Identified Proteins/Function | Locus | Fold Change | | | |
|---|---|---|---|---|---|
| | | Nipponbare | M202 | Cypress | Secano do Brazil |
| Leucine-rich family protein | OJ1288_C01.21 | **10.36** | **8.06** | 0.26 | 0.25 |
| Fructokinase-2 | Os08g0113100 | **2.23** | **1.84** | 0.77 | 0.65 |
| Phosphoribulokinase | Os02g0698000 | **5.90** | **4.82** | 0.57 | 0.29 |
| Nucleoside diphosphate kinase, Cell elongation process in coleoptile | Os07g0492000 | **7.67** | **11.06** | 0.99 | 0.83 |
| G-box factor 14-3-3a protein, wound-inducible WIN2 | Os08g0480800 | **6.59** | **14.87** | 0.26 | 0.37 |
| G-BOX Factor 14-3-3F protein | Os03g0710800 | 0.36 | 0.87 | **14.71** | **13.17** |
| Helix-loop-helix-like protein | Os07g0543000 | **7.94** | **29.22** | 0.67 | 1.01 |
| Putative glyoxalase 1 | P0669G10.7 | **4.42** | **3.84** | 0.63 | 0.13 |
| lipid-transfer protein 1 precursor (LTP 1) | Os12g0115100 | **10.13** | **6.44** | 2.94 | 0.75 |

Analysis was done with Skyline-daily 3.6.9 software, significant differences shown in bold fold change values.
